# Vascular smooth muscle cell metabolic reprogramming and phenotypic remodeling in atherosclerosis

**DOI:** 10.1038/s41420-025-02932-9

**Published:** 2025-12-30

**Authors:** Zhenyue Fu, Shuo Yang, Xindi Chang, Ping Liu, Yiru Wang

**Affiliations:** Department of Cardiology, Longhua Hospital, Shanghai University of Traditional Chinese Medicine, Shanghai, China

**Keywords:** Atherosclerosis, Mechanisms of disease

## Abstract

Metabolic reprogramming of vascular smooth muscle cells (VSMC) is emerging as a central driver of atherosclerotic plaque heterogeneity and instability. VSMCs undergo phenotypic remodeling into osteogenic, macrophage-like, foam cell–like, or pro-inflammatory states through metabolic reprogramming, which actively drives vascular calcification, lipid accumulation, and extracellular matrix degradation. In this review, we summarize the various phenotypes of VSMCs observed during AS development and describe potential molecular pathways linking metabolic reprogramming to phenotypic remodeling. We highlight key regulators, including glucose transporters, pyruvate dehydrogenase kinase 4, 6 - Phosphofructo - 2 - kinase/fructose - 2, 6 - bisphosphatase 3, pyruvate kinase M2, fatty acid synthase, homocysteine, etc., which integrate extracellular stimuli and intracellular metabolic changes to drive VSMCs fate decisions. In addition, we discuss how specific metabolic pathways interact with epigenetic and signaling networks to regulate VSMCs proliferation, apoptosis, calcification, foaming, and aging. Finally, we explore therapeutic opportunities for targeted metabolic regulators, including traditional Chinese medicine, Sirtuin 1 activators, ATP-Citrate Lyase inhibitors, statins, folic acid, etc., providing new strategies to stabilize plaques and slow the progression of AS.

## Facts

- VSMCs undergo metabolic reprogramming during the pathogenesis of atherosclerosis.
- Metabolic reprogramming of VSMCs can induce the transition from contractile to synthetic VSMCs, macrophage-like VSMCs, foam cell-like VSMCs, mesenchymal-like VSMCs, osteogenic-like VSMCs, myofibroblast-like VSMCs, fibroblast-like VSMCs, aging VSMCs and inflammatory VSMCs.
- The key substrates and enzymes in the metabolic reprogramming of VSMCs may be new targets for the treatment of atherosclerosis.

## Introduction

Atherosclerosis (AS) is a common chronic, inflammatory, and infiltrative cardiovascular disease that poses a serious threat to human health [1, 2]. Atherosclerotic plaques are characterized by the accumulation of cholesterol crystals in the arterial wall, a fibrous cap rich in extracellular matrix produced by vascular smooth muscle cells (VSMCs) and infiltration of immune cells (such as monocytes-macrophages, T cells, and mast cells) [3, 4]. Many evidence indicate that metabolic reprogramming is closely related to plaque stability. In high-risk plaques, glycolysis and pentose phosphate pathway (PPP) are enhanced, fatty acid oxidation (FAO) is reduced, and amino acid anaplerosis is increased [5–7]. Metabolic reprogramming of plaques provide clues to changes in the metabolic pattern of cells within the plaque.

VSMCs reside in the middle layer of the vascular system. It is a long-lived cell type that remains undifferentiated and exhibits high plasticity [8, 9]. It can regulate the vessels diameter and the extracellular matrix, and respond to changes in hemodynamics to regulate arterial compliance and elastic recoil [10]. Primary VSMCs exhibit a contractile phenotype with low proliferation potential, and highly express smooth muscle myosin heavy chain (SM-MHC), smooth muscle 22α (SM22α), and calmodulin [11]. When environmental cues and vascular homeostasis change, the morphology and function of VSMCs will undergo reversible changes. Among them, the dedifferentiation process of contractile-synthetic VSMCs is an important process in plaque formation [12]. Along with the decrease in contractile protein expression, the proliferation and migration ability are enhanced, the morphology changes from long spindle to short spindle or round, the myofilament density decreases, and ECM increases [13, 14]. With the advancement of lineage tracing technology and single-cell spatial sequencing technology, it has been found that VSMCs can also generate various alternative cell types such as osteogenic/chondrogenic VSMCs, myofibroblast-like cells, endothelial cells, macrophage-like foam cells, and inflammatory VSMCs to constitute unstable plaques with inflammation and ossification or stable plaque states with collagen matrix deposition and calcification [15–18].

Recent studies have found that changes in environmental cues during AS (ischemia, inflammation, etc.) not only directly regulate VSMC phenotype, but also indirectly regulate phenotype through metabolic reprogramming to generate a variety of replacement cells for adaptation [19–21]. Yet, few pharmacological strategies have been developed to target these metabolic pathways directly. Exploring the intersection between metabolism and phenotypic plasticity may provide novel therapeutic targets for AS. Therefore, we mainly describe the dynamic process of VSMCs metabolic rep programming and phenotypic remodeling during AS. More specifically, we first summarize the various phenotypes of VSMCs and their role in plaque construction and then focus on the regulatory mechanisms and molecular pathways of key substrates and enzymes in glucose, lipid, and protein metabolism on the phenotype of VSMCs. We also explored the potential therapeutic opportunities for targeted metabolism to rescue the phenotype of VSMCs. Finally, we summarize the emerging technologies of VSMCs dedifferentiation trajectory for further research.

## VSMCs phenotype classification and control switch

### VSMCs phenotype classification

**Fig. 1:**
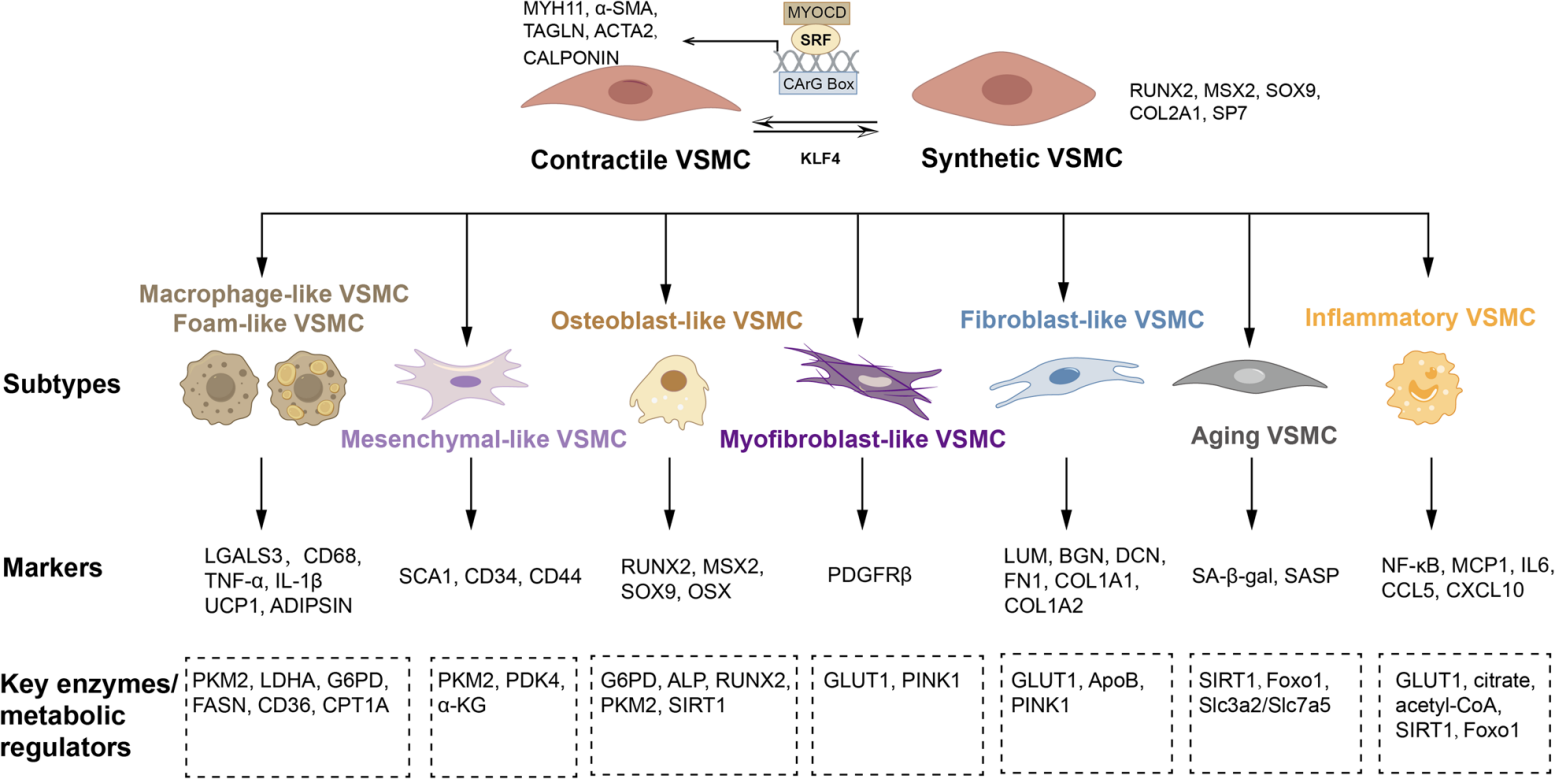
Phenotypic switch, dedifferentiation trajectories and markers of VSMC.

During the AS process, VSMCs lose their contractile phenotype markers, such as α-SMA and SM22α, while upregulating transitional markers, including fibrochondrocyte-related genes like Col1a1, Col1a2, and Col3a1, as well as complement and inflammation-related genes such as C3, CXCL12, and CXCL1. These transitional VSMCs can further differentiate into at least nine distinct phenotypic subtypes [22]. These phenotypes include not only the contractile VSMCs marked by MYH11, α-SMA, Calponin, TAGLN, and MYOCD [23, 24], but also synthetic VSMCs marked by RUNX2, MSX2, SOX9, COL2A1, and SP7 [25], macrophage-like VSMCs expressing LGALS3, CD68, TNF-α, and IL-1β [26, 27], foam cell-like VSMCs marked by UCP1 and ADIPSIN [28, 29], mesenchymal-like VSMCs marked by SCA1, CD34, and CD44 [30], osteogenic-like VSMCs marked by RUNX2, MSX2, SOX9, and OSX [31], myofibroblast-like VSMCs expressing PDGFRβ [6], fibroblast-like VSMCs expressing LUM, BGN, DCN, FN1, COL1A1, and COL1A2 [32, 33], aging VSMCs marker by SA-β-gal and SASP [34], and inflammatory VSMCs marked by NF-κB, MCP1, IL6, CCL5, and CXCL10 [35, 36]. The distribution and transition among these phenotypes dynamically in response to metabolic reprogramming, reflecting the trajectory of VSMC dedifferentiation [37]. (Fig. 1)

### Phenotypic switch of VSMCs

The phenotypic switch of VSMCs mainly includes myocardin (MYOCD) serum response factor (SRF) [38] and the Krüppel-like factor (KLF) family [39]. SRF switches the morphology of VSMCs according to extracellular cues [40]. The Q region associated with the N-terminal MADS domain of SRF binds to the CArG box (CC (A/T) 6GG) and MYOCD [41], forming an SRF-MYOCD-CArG box complex and activating the transcription of downstream target genes to regulate the contraction and differentiation of VSMCs [42]. The SRF-MYOCD-CArG box complex induce the transcription of VSMC-specific contractile protein genes, including SM22α, Calponin, SM-MHC, and Aortic Smooth Muscle Actin (ACTA2), to maintain the contractile phenotype. When the vascular wall is stimulated by hypoxia, mechanical damage, or platelet-derived growth factor (PDGF-BB), the transcription coactivator MYOCD and the nuclear transcription factor EIkI compete for the same binding site on the serum response factor, and the contractile phenotype-responsive SRF-MYOCD complex is converted to the synthetic phenotype-responsive SRF-ELK1 complex, prompting the VSMCs to transform from a contractile phenotype to a synthetic phenotype [43, 44]. At the same time, the binding of the SRF-MYOCD-CArG box complex may be antagonized by KLF4. KLF4 is a member of the Krüppel-like factors transcription factor family, with three conserved Cys2His2-type zinc fingers at the carboxyl terminus, which can specifically recognize and bind to GC-rich DNA sequences to regulate gene transcription [45]. KLF4 induces VSMC dedifferentiation through multiple mechanisms, including ubiquitination and degradation of myocardial proteins, post-translational modification (acetylation, phosphorylation, ubiquitination, and sumoylation modification), and destruction of the MYOCD/SRF complex [46]. KLF4 regulates myocardial protein expression through ubiquitin-dependent proteolysis and binds to the G/C repressor element, preventing SRF from binding to the CArG box and inhibiting the transcription of contractile proteins [47]. In addition, KLF4 also inhibits VSMC proliferation by binding to p53 and p21. KLF4 and Runt-related transcription factor 2 (RUNX2) synergistically enhance VSMC calcification [48].

## VSMCs glucose metabolism

VSMCs show markedly low metabolic efficiency: sustaining peak isometric tension requires only 1/300–1/500 the ATP required by fast skeletal muscle, yet their ATP is consumed 150-fold more slowly [49]. The VSMCs energy metabolism mainly rely on glycolysis and oxidative metabolism to supply actin-myosin cross-bridge activity. In a fully oxygenated resting state, 90% of glycogen is aerobic glycolysis to supply 30% of ATP, of which 69%-80% of metabolic equivalents are used to generate lactate [50]. During vasoconstriction, VSMCs inhibit exogenous glucose uptake and compensates for glycolytic flux through glycogenolysis. The energy supply mode is transformed into glycogenolysis and OXPHOS to meet the stability and efficiency of the energy supply during vasoconstriction [51]. However, aerobic glycolysis and oxidative metabolism are spatially compartmentalized. Glycogenolysis and glycolysis are carried out through independent enzyme pathways, and the glucose units produced by glycogenolysis are not mixed with the glycolytic pathway [52]. At the same time, VSMC’s creatine phosphate serves as a reserve of high-energy phosphate bonds. 2 μmol per gram of high-energy phosphate can supply 80% of the energy for a single contraction under non-steady-state conditions [49]. The decomposition of creatine phosphate provides rapid but short-lived energy, while glycolysis and oxidative metabolism provide sustained but slower energy. The complementary mechanism ensures that the energy needs of VSMCs are met under different conditions, like the Warburg effect of tumors [53]. The high reliance on low-energy-output aerobic glycolysis by resting VSMCs may be attributed to the fact that glycolysis-derived ATP primarily maintains intracellular Na⁺/K⁺ homeostasis, whereas ATP from oxidative metabolism supports contractile function, and the high lactate production from glycolysis enhances mitochondrial reserve capacity [54].

**Fig. 2:**
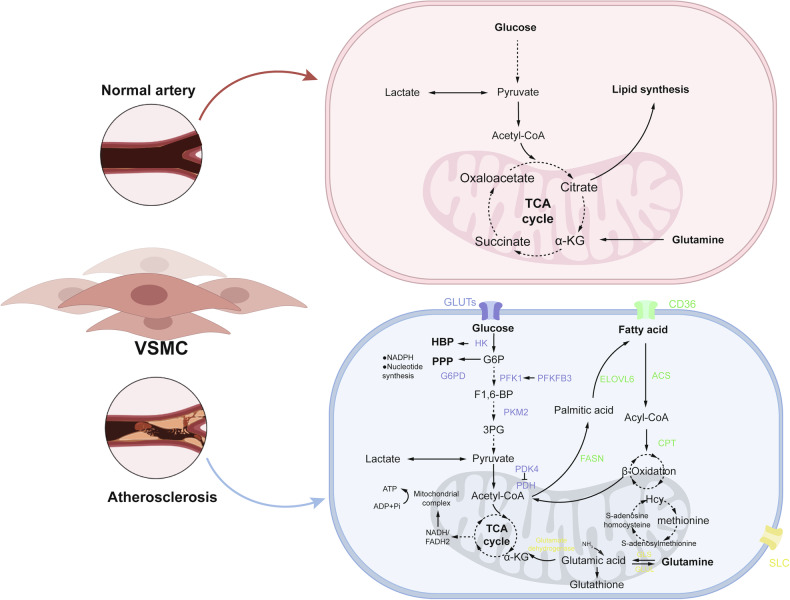
Metabolic reprogramming of VSMCs in AS. Under physiological conditions, VSMCs mainly rely on glycolysis and the tricarboxylic acid (TCA) cycle to maintain energy homeostasis. In AS, VSMCs undergo profound metabolic reprogramming involving three major pathways: 1. Glucose metabolism: Enhanced glycolytic flux is supported by key enzymes, including HK (hexokinase), G6PD (glucose-6-phosphate dehydrogenase), PFK1 (phosphofructokinase-1), PFKFB3 (6-phosphofructo-2-kinase/fructose-2,6-bisphosphatase 3), and PKM2 (pyruvate kinase M2), which channel glucose toward biosynthetic and redox pathways. 2. Fatty acid metabolism: VSMCs increase fatty acid uptake, activation, and utilization, mediated by ELOVL6 (elongation of very long chain fatty acids protein 6), ACS (acyl-CoA synthetase), CPT (carnitine palmitoyltransferase), and FASN (fatty acid synthase), promoting both β-oxidation and lipogenesis. 3. Amino acid metabolism: Uptake through SLC transporters and conversion by GLS (glutaminase) supply α-ketoglutarate to the TCA cycle and contribute to glutathione synthesis, thereby supporting anabolic processes and antioxidant defense. These metabolic reprogramming provide bioenergetic and biosynthetic substrates for VSMCs proliferation and phenotypic remodeling, ultimately accelerating atherosclerotic plaque development.

VSMCs are stimulated by various pathological factors (such as mechanical stress changes and inflammatory factors), and the glucose metabolism pattern is converted to the promotion of glycolytic flux, the reduction of PPP and mitochondrial metabolism, accompanied by phenotypic conversion to synthesis, osteogenic, foam cell, and inflammatory types. A variety of enzymatic reactions play a role in the metabolic reprogramming, such as hexokinase(HK), pyruvate dehydrogenase kinase 4 (PDK4), PFKFB3 (6-phosphofructo-2-kinase/fructose-2,6-bisphosphatase 3), PKM2 (pyruvate kinase M2 type), and this process is regulated by peroxisome proliferator-activated receptor gamma (PPARγ), advanced glycation end products (AGEs), glucose transporters (GLUTs), Sirtuin 1 (SIRT1), Mammalian target of rapamycin (mTOR), and hypoxia-inducible factor 1-alpha (HIF-1α), etc. (Fig. 2)

### Glycolysis

#### GLUTs family

The transport of extracellular glucose into cells through GLUTs on the cell membrane is the first step in VSMCs glucose metabolism. A total of 14 GLUTs have been identified, which can be divided into the following three categories: Class I includes GLUT1-4 and GLUT14, Class II includes GLUT5, 7, 9, and 11, and Class III includes GLUT6, 8, 10, 12, and 13 [55]. In proliferative VSMCs, GLUT1 is the isoform with the highest relative abundance (~43%) of GLUT family mRNA, has a high affinity for glucose (K_m_=3 mM), and can increase the uptake of the radiolabeled glucose analog 2-[3H]-deoxy-D-glucose by 3 times [56, 57]. Overexpression of GLUT1 can increase cFLIP (a pro-survival molecule that inhibits caspase activity) to promote the anti-apoptotic and pro-proliferative effects of VSMCs. Treatment with non-metabolizable glucose analogs and the glycolysis inhibitor 2-deoxyglucose or knockdown of cFLIP abolished the above effects, suggesting that GLUT1 exerts its anti-apoptotic effects through glycolysis [58].

In metabolic-syndrome-induced atherosclerotic lesions, VSMCs near the plaque showed elevated GLUT1 and accumulated glucose, sorbitol, and glyoxal, indicating that GLUT1 drives both glycolytic and polyol flux. In SM22α promoter–driven, VSMC–specific GLUT1 overexpressing mice (SM-GLUT1 mice) fed a high-fat, high-sucrose diet containing 0.15% cholesterol, an increase in the expression of synthetic phenotype–associated genes (Has1 and Has3) was observed, along with enhanced recruitment of Ly6C⁺⁺ monocytes and upregulation of CCL2 expression. These findings indicate that GLUT1 overexpression simultaneously promotes VSMC phenotypic remodeling toward a synthetic state and monocyte recruitment, thereby forming a proliferative and inflammatory positive feedback loop that accelerates AS progression [59]. Increased VSMC glucose uptake, glutathione, and haptoglobin were also observed in SM-GLUT1 mice subjected to mechanical arterial injury, indicating that GLUT1 overexpression simultaneously promotes aerobic glycolysis and increased pentose phosphate flux. It induces MCP-1 mediated inflammatory signals and pSMAD2/3 mediated profibrotic signal pathway activation, increasing the expression of the matrix gene decorin from glucose response elements and reducing fibronectin, ultimately leading to VSMC proliferation and hypertrophy [60]. In addition, upregulation of GLUT1 altered the glucose metabolism pathway in [U-13C] labeled VSMCs, causing glucose to participate more in non-oxidative metabolism (such as glycolysis to produce lactate) and oxidative metabolism (such as entering the TCA cycle). Overexpression of GLUT1 increased the phosphorylation level of glycogen synthase kinase 3β (GSK3β), inhibiting VSMC apoptosis induced by serum starvation or Fas ligand stimulation, and significantly increasing the proportion of VSMCs in the S, G2, and M phases. This suggests that GSK3β may be a key mediator connecting metabolic signals to the fate of VSMCs [61].

GLUT4 is an insulin-responsive glucose transporter with a slightly lower affinity for glucose (K_m_=5 mM). It accounts for a very low proportion of proliferating and differentiating VSMCs (~0.2% and ~0.02%). However, under insulin stimulation, the membrane translocation of GLUT4 can increase the efficiency of glucose transport by about 10–20 times, mediating 50% of the basal glucose uptake [62, 63]. In VSMCs stimulated by PDGF-BB, the expression of GLUT4 increased in a time-dependent manner, accompanied by an increase in the ATP/ADP ratio and L-lactate concentration, an increase in proliferating cell nuclear antigen (PCNA), and a decrease in insulin sensitivity, indicating that GLUT4 promotes glucose metabolism to induce VSMC proliferation and insulin resistance. Immunofluorescence staining showed that GLUT4 was transferred from the cytoplasm to the cell periphery and co-localized with polymerized cortical actin to overcome insulin resistance. Knockdown of SM22α enhances GLUT4 membrane translocation by promoting actin dynamics and cortical actin polymerization to promote VSMC proliferation and neointimal formation [64].

#### HK

HK is the first rate-limiting enzyme in the glycolysis process. It catalyzes the phosphorylation of glucose to produce glucose-6-phosphate, thereby converting glucose into intermediates in the intracellular metabolic pathway [65]. When blood vessels are damaged, the activities of HK and lactate dehydrogenase increase, promoting VSMC phenotypic remodeling (proliferation, migration, dedifferentiation). Myotubularin-related protein 7 (MTMR7) is a member of the phosphatase family and plays an anti-proliferative role in a variety of cells including pulmonary artery smooth muscle cells, spermatogonial stem cells, and myoblasts. MTMR7 overexpression suppresses mTORC1 activation by phosphorylating p62 and dissociating the mTORC1 component Raptor, resulting in decreased HK activity and subsequent VSMC phenotypic remodeling and vascular repair [66]. The above results demonstrate that MTMR7 targets p62/mTORC1 to regulate the glycolysis and VSMC phenotypic remodeling, providing metabolic insights into the anti-proliferative effect of MTMR7 [67–69].

#### PFKFB3

PFKFB3 is an enzyme that synthesizes fructose-2,6-bisphosphate (Fru-2,6-P2). Fru-2,6-P2 is a potent allosteric activator of phosphofructokinase-1 (PFK-1), a key rate-limiting enzyme in the glycolytic pathway, which can accelerate the glycolytic process and lactate production [70, 71]. Recent studies have found that PFKFB3-mediated VSMC glycolysis is closely related to the metabolic homeostasis of VSMCs and accelerates vascular diseases such as AS [72], pathological angiogenesis [73], and pulmonary hypertension [74]. PFKFB3-induced lactate production can activate calpain2 through an extracellular signal-regulated kinase 1/2 (ERK1/2)-dependent mechanism. Activation of calpain2 can lead to phosphorylation of AKT at Ser473 and Thr308 site to promote the synthesis of collagen I and VSMC proliferation [75]. Concurrently, PFKFB3-driven accumulation of lactate and pyruvate activates the FOXO3 signaling pathway, upregulating osteogenic genes such as alkaline phosphatase (ALP), osteocalcin, and osteoprotegerin, and ultimately facilitating the osteogenic dedifferentiation of VSMCs [76]. Multiple tyramide signal amplification staining found that approximately 10-20% of α-SMA-positive and KLF4-positive cells in AS lesions expressed CD123 and CD68. Overexpression of KLF4 upregulates Eukaryotic Elongation Factor 1 Alpha 2 (eEF1A2), which interacts through the circCTDP1–eEF1A2 complex to facilitate the translation of PFKFB3. At the same time, the phosphorylation of Ser727 and the acetylation of Lys685 of STAT3 were enhanced, and the phosphorylation of AKT was also significantly increased. It indicates that the promotion of glycolysis driven by KLF4-PFKFB3 can regulate the conversion of contractile VSMCs into pDC-like VSMCs through the AKT pathway [77].

In VSMCs from carotid artery stenosis lesions, upregulation of HIF-1α and PFKFB3 was observed, along with an increase in glycolysis, maximum glycolytic capacity, and glycolytic reserve. Additionally, there was an elevation in the expression of cell cycle proteins (pH3, PCNA, and cyclin D1) and a decrease in contractile markers (α-SMA, SM22α, SMMHC, and MLCK). These changes were associated with an increase in the downstream p-p70S6K of mTORC1 signaling. mTORC1 is a cell stress sensor that captures a variety of extracellular cues (energy conversion, growth factors) to regulate cell growth and metabolism [78]. At the same time, upregulation of PFKFB3 builds a carbon atom-sharing bridge between glycolysis and fatty acid synthesis. Acetyl CoA and citrate produced by glycolysis provide fuel for de novo fatty acid synthesis [79].

#### PDK4

PDK4 is a key mitochondrial matrix enzyme in the oxidative decarboxylation process of pyruvate. It regulates the activity of the pyruvate dehydrogenase complex (PDC) by phosphorylating and dephosphorylating PDCE1α (a subunit of PDC) [80]. PDK4 phosphorylates and inactivates pyruvate dehydrogenase (PDH), preventing pyruvate from entering the mitochondria to participate in the TCA cycle, and forcing VSMCs to rely more on the glycolysis to generate energy [81]. Numerous studies have demonstrated that PDK4 plays a crucial role in determining the VSMC osteogenesis. In the high-concentration Pi-induced VSMC calcification model, overexpression of PDK4 located in the mitochondria and cytoplasm increased the phosphorylation level of SMAD1/5/8 and promoted its nuclear translocation, enhancing the activity of Runx2 and ALP promoters downstream of Bone Morphogenetic Protein 2 (BMP2) through the BMP2/SMAD pathway, inducing VSMC osteogenesis. Overexpression of PDK4, localized in both the mitochondria and cytoplasm, increases the phosphorylation of SMAD1/5/8 and promotes their nuclear translocation, enhances the transcriptional activity of downstream Runx2 and ALPL promoters via the BMP2/SMAD pathway, ultimately inducing VSMC osteogenesis. At the same time, PDK4 overexpression induces phosphorylation-mediated inactivation of PDC, resulting in mitochondrial respiratory dysfunction and mitochondria-dependent cell apoptosis, further promoting VSMC calcification [82].

HIF-1α is a member of the hypoxia-inducible factor family [83]. Under hypoxic conditions, proline hydroxylase cannot negatively regulate the stability and ubiquitination degradation of HIF-1α through oxygen-dependent hydroxylation modification, resulting in HIF-1α accumulation and translocation to the nucleus and binding to HIF-1β to form a complex, followed by the recruitment of coactivators (CBP/P300) at the hypoxia response element (HRE) to upregulate the transcription of glucose transporters and glycolytic enzymes to promote anaerobic glycolysis [84, 85].

Meanwhile, hypoxia activates the HIF-1α/PDK4 axis, which inhibits PDH activity and elevates mitochondrial membrane potential(MMP), suppressing the TCA cycle while enhancing glycolysis and lactate production, ultimately driving VSMC cell cycle progression and proliferation [86]. SIRT6 is a member of the highly conserved class III histone deacetylas [87]. Overexpression of SIRT6 inhibits the transcriptional activation of PDK4 and HIF-1α-dependent glycolytic genes, leading to G1 phase arrest of VSMCs and blocking cell cycle progression [86]. AGEs are a group of heterogeneous compounds generated by non-enzymatic glycosylation of proteins, lipids, or nucleic acids [88]. AGEs increase HIF-1α and PDK4 transcription and translation in a dose-dependent manner and induce nuclear translocation of HIF-1α. PDK4 can upregulate RUNX2 and ALP activity, increase calcium deposition and calcified nodule formation, and promote the transformation of VSMC osteogenesis [89]. The upregulation of HIF-1α/PDK4 by AGEs seems to indicate that AGEs should promote glycolysis. However, AGEs intervention reduces the expression of key glucose metabolic enzymes—including HK, LDH, IDH, and G6PD—along with a decline in mitochondrial respiratory capacity. Moreover, lactate production and glucose consumption decrease in a dose-dependent manner, indicating that AGEs suppress glycolysis, aerobic metabolism, and PPP during VSMC calcification [89]. In addition, AGEs can enhance the autophagy pathway by upregulating the HIF-1α/PDK4 pathway to induce VSMC calcification, while the PDK4 inhibitor DCA can reverse it [90]. The immunogen in AGEs, Nε-carboxymethyl-lysine, upregulates PDK4 expression through the oxidative stress pathway and induces VSMC osteogenesis [91]. The above results suggest that AGEs may regulate the formation of VSMC osteogenesis through HIF-1α/PDK4, which may depend on autophagy and oxidative stress. Although extensive studies have explored the role of AGEs/PDK4 signaling in glucose metabolism, conflicting conclusions have been reported. These discrepancies may arise from differences in experimental conditions, such as glucose concentration, stimulation time, or the metabolic phenotype of VSMCs. Future investigations should employ standardized models and multi-omics approaches to clarify whether AGEs/PDK4 exerts a context-dependent regulatory role in metabolic reprogramming during vascular calcification and AS.

#### PKM2

PKM2 is a key enzyme in the last step of glycolysis, converting phosphoenolpyruvate to pyruvate and supplying ATP [92]. PKM2 has three conformations including inactive monomer, dimer, and tetramer. The enzymatically active tetramer (tet-PKM2) has a high affinity for glycolytic substrates and enzymes to promote the conversion of glucose to pyruvate or lactate, while the transcriptionally active dimer (di-PKM2) translocates to the nucleus and acts as a nuclear transcriptional coactivator that regulates gene expression while allowing the accumulated glycolytic intermediates to generate cellular building block components (such as nucleotides and lipids) through the phospholipid synthesis and amino acid synthesis [93–96].

Post-translational modification and tetramer-to-dimer ratio adjustment of PKM2 are associated with VSMC metabolic reprogramming, inducing the shift from a contractile phenotype to anabolic and osteogenic VSMCs. Under the stimulation of ox-LDL or PDGF-BB, PKM2 expression was upregulated in a concentration- and time-dependent manner, accompanied by increased glucose uptake, lactate, and ATP production. It indicates that PKM2-dependent glycolysis increases the proliferation, migration, and synthetic capacity of VSMCs [97]. In PKM2^fl/fl^ SM-MHC-CreERT2 mice, SM22α and SM-MHC expression were increased, while vimentin and osteopontin expression were decreased, reflecting the association between glycolysis inhibition and the maintenance of VSMC contractile phenotype. Further studies have found that PDGF-BB can induce PKM2 tyrosine 105 phosphorylation, promote PKM2 nuclear translocation, and inhibit its tetramerization. Di-PKM2 interacts with STAT3 and β-catenin, regulates the transcription of MEK5, Cyclin D1, GLUT1, and LDHA, induces aerobic glycolysis, and promotes the proliferation, migration, and osteogenesis of VSMCs [98].

Crotonylation of PKM2 at the K305 site can promote the formation of dimers after nuclear translocation and promote the expression of glycolysis-related proteins GLUT1 and LDHA, increase glycolysis intermediates such as pyruvate and fructose-1,6-bisphosphate, and induce upregulation of PCNA and OPN, and downregulation of CNN1, α-SMA, and Transgelin [99]. The K305R mutation reduces the extracellular acidification rate (ECAR) and lactate production, and the proliferation activity of VSMC is weakened along with the inhibition of glycolysis.

HnRNPA1 is a key regulator of PKM2 mRNA splicing, which can promote PKM2 expression and glycolysis. The C-terminus of prohibitin 2 can interact with hnRNPA1 to inhibit PKM2 splicing and glycolytic reprogramming, thereby maintaining the contractile phenotype of VSMC and inhibiting neointimal formation [100].

#### G6PD

G6PD is a key enzyme in PPP [101], catalyzing the oxidation of G6P to 6-phosphogluconolactone and reducing NADP⁺ to NADPH to maintain the reduced state of glutathione and provide pentose for nucleic acid biosynthesis [102]. Although there are few studies on the role of G6PD in promoting VSMC phenotypic conversion during AS, its role in regulating VSMC phenotype has been confirmed in pulmonary hypertension, acute pulmonary hypoxia, metabolic syndrome, and hypertension [103–105]. The expression of contractile genes in VSMCs, including MYH11, CNN1, and SM22-α, was upregulated following treatment with 6-aminonicotinamide (a G6PD inhibitor). Similarly, G6PD-deficient mice exhibited increased expression of VSMC contractile genes, such as SRF-MYOCD–dependent Lmod1, Cnn1, Kcnmb1, and Actg2. Enrichment analysis showed that G6PD deficiency upregulates genes related to translation, actin filament-based processes, cell adhesion, and cytoskeletal structure, and downregulates genes related to homeostatic processes, lipid transport, and lipid localization [106]. Under hypoxic stimulation, the activity of G6PD increased by about 1.5 times, promoting the metabolism of the PPP and increasing the level of NADPH. At the same time, G6PD formed a complex with TxR-1 and PKG to inhibit PKG activity and promote the expression of SM22α and SM-MHC [107]. PKG is a cGMP-dependent protein kinase, indicating that PKG may be a potential target for G6PD to promote VSMC proliferation.

Further studies have found that G6PD can exert its effects through PKG-dependent or -independent pathways. In the PKG-independent pathway, 6-aminonicotinamide can directly stimulate the expression of MYOCD/miR-143 and downregulate the synthetic phenotype-responsive SRF-ELK1 complex to maintain the contractile phenotype. In the PKG-dependent pathway, 6-aminonicotinamide increases the expression and activity of PKG1α, upregulates the expression of miR-1, and reduces the expression of KLF4 to maintain the contractile phenotype [106]. Interestingly, during acute hypoxia, G6PD can activate the RhoA-ROCK signaling pathway, which drives actin cytoskeletal remodeling, increases smooth muscle calcium sensitization and myosin 20KD light chain phosphorylation, and promotes contraction of PASMCs [108].

Voltage-dependent anion channel protein 1 (VDAC1) is a multifunctional channel protein located in the outer membrane of mitochondria [109]. Immunoprecipitation revealed that the N-terminal domain of G6PD interacts with VDAC1 and competes with Bax for binding to VDAC1, alleviating VSMC apoptosis by reducing VDAC1 oligomerization and maintaining the synthetic phenotype of VSMC [110]. In addition, SM22α can synergistically enhance G6PD activity, stimulate the PPP to increase NADPH production, maintain GSH homeostasis, and promote VSMC proliferation and migration [111].

### Warburg-like effect and lactate accumulation

Lactate is the end product of anaerobic glycolysis, which is produced by the nicotinamide adenine dinucleotide-dependent reduction of pyruvate catalyzed by lactate dehydrogenase-A (LDHA) [112]. In a lactate-rich microenvironment, the number of live cells of hiPSC-VSMC and HA-SMCs increased by about 55–65%, and their morphology changed from a regular spindle shape to an irregular flat shape, accompanied by a decrease in contractile markers such as α-SMA, SM-MHC, and calponin and an increase in synthetic markers such as vimentin, Collagen I, MMP-9, and OPN [113].

In vitro and in vivo experiments have shown that lactate can control the phenotypic remodeling of VSMCs by regulating mitochondrial metabolism, mitochondrial dynamics, and mitochondrial autophagy. There are two main pathways of mitochondrial autophagy: the receptor-independent pathway mediated by PINK1-Parkin and the receptor-dependent pathways such as NIX, BNIP3, and FUNDC1 [114–116]. In addition, Drp1-mediated mitochondrial fission also plays an important role in the process of alternative mitochondrial autophagy [117]. After exogenous lactate intervention, primary VSMCs lost their contractile phenotype and differentiated into osteoblasts, accompanied by the increased opening rate of mitochondrial permeability transition pore, a significant decrease in MMP and mitochondrial energy generation capacity, and impaired mitochondrial biogenesis. At the same time, LC3-II and BNIP3 levels decreased, and the co-localization of LC3-II and LAMP-1 decreased. Lentivirus-mediated BNIP3 overexpression reversed the above results. It indicates that lactate inhibits the protective autophagic flow and the fusion of autophagosomes and lysosomes in the BNIP3 pathway to regulate VSMC osteogenesis [118]. Further studies have found that lactate activates Drp1-related mitochondrial fission and blocks the protective autophagy of BNIP3 through the NR4A1/DNA-PKcs/p53 signaling pathway, leading to mitochondrial structural destruction, decreased MMP, and impaired respiratory function, ultimately exacerbating VSMC osteogenesis and vascular calcification [119]. NR4A1 belongs to the orphan nuclear receptor NR4A family and is a transcription factor highly expressed in VSMCs [120]. When VSMCs are damaged, NR4A1 blocks the binding of DNA-PKcs to the Ku80-Ku70 heterodimer, inhibits the DNA double-strand break sensing process, promotes DNA-PKcs to phosphorylate p53 at Ser15 site and induces activation of p53-dependent apoptosis [121, 122]. In the co-culture system of VECs and VSMCs under high glucose and high phosphorus microenvironment, VECs secrete a large amount of lactate, which directly leads to the transformation of VSMC osteogenesis. Lactate leads to overactivation of Drp1-related mitochondrial fission and inhibition of PINK/Parkin-mediated mitophagy through the PARP1/POLG/UCP2 axis [123]. Poly (ADP-ribose) polymerase 1 (PARP1) is a nuclear DNA damage sensor that is recruited to the damaged site after DNA strand breakage to initiate co-condensation with broken DNA to initiate DNA repair [124]. The POLG gene encodes the catalytic subunit of mtDNA polymerase, and its mutation can lead to mtDNA loss or depletion [125]. Lactate induces PARP1 to translocate from the nucleus to the mitochondria, where it binds to POLG to directly inhibit mtDNA synthesis, mitochondrial respiration, and OXPHOS. At the same time, PARP1/POLG signaling upregulates UCP2, a member of the mitochondrial uncoupling protein family that can inhibit PINK1/Parkin-mediated autophagy [123]. Therefore, lactate accumulation caused by metabolic disorders primarily affects mitochondrial homeostasis to regulate VSMC phenotype and accelerate the progression of AS.

TNF receptor-associated protein (TRAP1) is a mitochondrial homolog of the heat shock protein 90 chaperone family that regulates the transition between OXPHOS and glycolysis [126]. The interaction between TRAP1 and the rate-limiting enzyme PFK1 promotes aerobic glycolysis, leading to increased lactate production. Lactate promotes lactic acidification of histone H4 lysine 12 (H4K12la) by downregulating histone lysine delactase, inducing the enrichment of H4K12la in the SASP promoter region and SASP transcriptional activation to promote VSMC senescence. In TRAP1-knockout rats, acetyl-CoA and citrate were increased, and the expression levels of senescence markers (P53, P21, and P16) and H4K12la in VSMCs were decreased, while lipid accumulation, plaque area, and necrotic core size in the aorta were reduced [127]. It suggests that TRAP1-mediated lactate accumulation can promote cell senescence through histone modification pathways.

LDHA catalyzes the reduction of pyruvate to lactate through a nicotinamide adenine dinucleotide-dependent pathway [128]. When blood vessels are damaged, LDHA-mediated metabolic promotion can increase VSMC proliferation and migration to repair blood vessels. After treatment with fetal bovine serum or PDGF, glucose uptake, lactate production, and ATP production increased, ECAR and OCR increased, and VSMC proliferation and migration increased. Oxamate (LDHA inhibitor) and siRNA knockdown of LDHA inhibited the metabolic promotion and differentiation of VSMCs [129].

### Hexosamine Biosynthetic Pathway (HBP)

The HBP is a branch of glycolysis that branches from the downstream of HK. It integrates the metabolism of glucose, glutamine, uridine triphosphate, and acetate, and ultimately produces uridine diphosphate N-acetylglucosamine (UDP-GlcNAc) [130]. UDP-GlcNAc is transferred to the corresponding target protein serine/threonine residues by O-GlcNA transferase, resulting in oxygen-linked N-acetylglucosamine (O-GlcNAc) modification, which is coupled with post-translational modifications to regulate a variety of biological processes in the body [131].

The nuclear-encoded mitochondrial protein polymerase δ-interacting protein 2 (Poldip2) can bind to the p50 subunit of DNA polymerase, controlling the lipoylation and activation of PDH and α-ketoglutarate dehydrogenase (αKGDH) during metabolism [132]. It also regulates the cell cycle [133], cell migration [134], focal adhesion turnover, and force polarization [135] of VSMCs and participates in the process of AS. When Poldip2 is deficient, it induces protein O-GlcNAcylation and inhibits the ubiquitin-proteasome system through metabolic reprogramming (TCA cycle inhibition, glycolytic activity, increased HBP activity), resulting in the accumulation of polyubiquitinated proteins in the nucleus to maintain the stability of SRF, ultimately leading to VSMC differentiation [136].

### TCA cycle

Citrate is mainly localized in mitochondria, enters the classical TCA cycle through aconitase. Partial citrate is partially translocated to the cytoplasm through the mitochondrial citrate carrier SLC25A1, and then regenerates mitochondrial oxaloacetate under the cleavage induced by ATP citrate lyase to complete the non-classical TCA cycle process to regulate chromatin dynamics [137, 138]. Ankylosing kinase protein (ANK) mediates the output of intracellular citrate. Its deficiency can lead to intracellular citrate accumulation and the generation of acetyl-CoA in abdominal aortic aneurysm [139]. In VSMC-specific ANK knockout mice, the levels of citrate and acetyl-CoA increased significantly, which in turn promoted the acetylation of histones H3K23ac, H3K27ac, and H4K5ac, inducing the mRNA expression of pro-inflammatory factors (CCL2, IL-6, and MMP9) and the pro-inflammatory phenotype conversion of VSMCs. RNA sequencing showed that ANK deficiency and citrate accumulation activated the transcription of genes related to inflammation and energy metabolism through epigenetic mechanisms, especially the TNF signaling pathway, which was the pathway where these differentially expressed genes were co-enriched with histone acetylation sites [140]. This suggests that citrate-mediated histone acetylation modification plays a role in the pro-inflammatory phenotype conversion of VSMCs.

Aconitase is the catalytic enzyme of the first step of TAC [141]. Its inhibition damages mtDNA, resulting in blocked ATP synthesis and oxidative stress, hindering VSMC proliferation, inducing elastic fiber degradation, and collagen deposition. Supplementation of α-ketoglutarate, an intermediate of citrate metabolism, rescued the abnormal proliferation of VSMCs by increasing the mitochondrial ETC complex [142].

### OXPHOS

OXPHOS occurs on the inner membrane of mitochondria, completing proton cycling and ATP production through complexes I, II, III, and IV of the electron transport chain and ATP synthase [143]. Mitochondrial function and bioenergetic metabolism are closely related to the phenotypic remodeling of VSMCs. In the early stages of VSMC dedifferentiation, the basal respiratory capacity of complex I is enhanced, and the coupling between oxidation and phosphorylation is weakened, leading to increased mitochondrial transmembrane proton leak and ROS to promote the development of AS. As VSMCs differentiate, their dependence on lipid OXPHOS increases, and the expression of complexes II and IV is upregulated to support proliferation, migration, and synthesis and secretion of extracellular matrix [144]. In addition, the mitochondrial complex I inhibitor rotenone and the mitochondrial complex III inhibitor azoxystrobin target PI3K/AKT to inhibit VSMC proliferation and migration, thereby alleviating early and late AS in ApoE^-/-^ mice [145].

PINK1/Parkin plays a role as a biosensor in VSMC OXPHOS by maintaining Ca^2+^ balance, promoting mitochondrial biogenesis, promoting the transmission of the mitochondrial respiratory chain, and regulating mitochondrial autophagy [146]. In addition, PINK1 and mitoHKII co-localize on the inner membrane of mitochondria. They can induce Parkin to initiate ubiquitination through two independent pathways: Ser65 phosphorylation activation of Parkin or direct recruitment to promote mitochondrial autophagy. This mechanism provides a coupling for OXPHOS damage and compensatory glycolysis during cell damage [147]. The latest studies have found that the regulation of Parkin by PINK1 and HKII is not completely independent, which provides new insights into the metabolic flexibility between glycolysis and oxidative metabolism. In the microdissection and phenotypic analysis of human carotid artery plaques, high levels of SMA^+^ and MHC^+^ VSMCs and high expression of PINK1 were observed in the plaque cap [148]. When mtDNA is damaged, the expression of PINK1 increases, and complex II and IV-dependent respiration is significantly impaired. After the respiratory chain uncoupler caused the loss of MMP, the level of PINK1 stabilized and co-localized with TOM20 to phosphorylate and activate AMPK, and the energy compensation sensing mechanism was activated, which was manifested by increased expression of HK, increased glycolytic flux, and significantly increased expression of GAPDH, LDH, and PDK [148]. After inhibiting mitochondrial respiration, PINK1-KO VSMCs had impaired OXPHOS, reduced metabolic conversion capacity, and metabolic flexibility, manifested by significantly reduced OCR, complex I and IV-dependent respiratory defects, and limited glycolytic switching. Mitochondrial dysfunction and energy deficiency lead to reduced VSMCs, reduced fibrous cap thickness, and increased plaque vulnerability in atherosclerotic plaques of PINK1-KO mice [149].

## Lipid metabolism

FAO is a high oxygen consumption and high energy production metabolic pathway, including three steps: activation, transport, and β-oxidation, which ultimately produces FADH₂, NADH, and acetyl-CoA [150]. In VSMCs, the activation process of FAO is combined with glucose metabolism. The energy produced by aerobic glycolysis can be provided by FAO, and the activity of glycolysis is regulated by the feedback of FAO metabolites [151]. VSMCs are more dependent on FAO energy production during proliferation, with an increase in O_2_ consumption of 30% and a decrease in glucose oxidation and lactate production of 20% and 64%, respectively [152]. The phenotypic remodeling of VSMCs occur during fatty acid synthesis, elongation, and β-oxidation of fatty acid, and is also affected by various exogenous lipids.

### Fatty acid synthesis and elongation

Glycolysis generates acetyl-CoA and citrate, which are the energy sources for the de novo synthesis of FAS from fatty acids to supply the endoplasmic reticulum and Golgi apparatus required for cell proliferation [153]. Glycolysis not only provides a carbon source for FAS but also upregulates a variety of lipogenic enzymes, including fatty acid synthase (FASN), acetyl-CoA carboxylase, triglyceride synthase, acyl-CoA synthetase, enoyl-CoA hydratase, and 3-Ketoacyl-CoA synthase. FASN knockout or inhibition can inhibit PDGF-induced VSMC proliferation and migration [79]. FASN can also mediate the activation of the KLF4 by affecting cholesterol esterification and efflux pathways, promote the dedifferentiation of VSMCs into foam cells. In FASN-deficient or silenced VSMCs, cholesterol treatment increases the expression of the cholesterol efflux protein ABCA1 and reduces cholesterol-induced KLF4. In contrast, supplementation with palmitate, a major product of FASN, aggravates intracellular lipid accumulation and promotes the formation of a foam cell–like phenotype [154].

Elongation-of-very-long-chain-fatty acids 6 (ELOVL6) mediates the elongation reaction of palmitate (C16:0) to stearate (C18:0) [155]. The latest study found that Elovl6 co-localizes with α-SMA -positive cells during vascular injury. In Elovl6^-/-^ mice, the formation of new intima is significantly reduced, the number of Ki-67-positive cells and the expression of α-SMA and SM22α are reduced. This process is accompanied by increased palmitate levels, decreased oleic acid levels, and increased ROS production. ROS phosphorylates AMPK and allows KLF4 to bind to cell cycle proteins p53 and p21 to inhibit VSMC cell cycle [156].

### Fatty acid β-oxidation

Extracellular mechanical stress not only induces a shift in VSMC metabolism from OXPHOS to glycolysis, accompanied by FAO dysregulation and phenotypic remodeling [157, 158]. Cyclic stretching of arteries activates the nuclear mechanical sensor cPLA2 to produce arachidonic acid (ArAc). This process promotes proteasomal degradation of the transcription factor YY1, thereby downregulating carnitine palmitoyltransferase 1B (CPT1B). As a result, FAO is suppressed, causing accumulation of intracellular long-chain fatty acids and reduced palmitate oxidation activity, ultimately enhancing VSMC proliferation and migration [158].

Malonyl-CoA decarboxylase (MCD) is an endogenous inhibitor of CPT1 and a key regulatory enzyme of FAO [159]. In MCD knockout mice, FAO and OXPHOS to glycolysis metabolism is inhibited, accompanied by inhibition of VSMC proliferation [160]. cPLA2 can hydrolyze the ester bond at the SN-2 site of glycerophospholipids to produce fatty acids and lysophospholipids, directly providing substrates for FAO [161]. Its subtypes IIA/V/X can hydrolyze cholesterol family members HDL and LDL. Before and after hydrolysis, HDL and LDL can synergistically promote VSMC mitosis, while inducing VSMCs to release eicosanoid family members (prostaglandin E2 and leukotriene B4) to further promote AS [162]. Adipokine stimulation upregulates CD36, a key fatty acid transporter in VSMCs, resulting in enhanced fatty acid uptake and lipid deposition, ERK pathway activation, and subsequent VSMC proliferation and inflammatory responses [163].

### Exogenous lipids

In the 1990s, research teams found that non-esterified fatty acids, including oleic acid, linoleic acid, and their metabolites, can promote VSMC mitosis through the protein kinase C pathway and lead to vascular remodeling [164–166]. The latest studies have found that non-esterified fatty acids including oleic acid, linoleic acid, α-linolenic acid, and docosahexaenoic acid and lipoxygenase (LO) products including 15(S)-hydroxyeicosatetraenoic acid can lead to a dose-dependent and time-dependent increase in the phosphorylation of cAMP response element binding protein (CREB), thereby promoting VSMC migration and the formation of new endothelial cells [167, 168]. Interestingly, cAMP activation of CREB has a dual effect on VSMC mitosis. When cAMP activation of CREB is CRTC2/3-dependent and serine-133-independent, it inhibits VSMC mitosis. When cAMP activation of CREB is serine-133-dependent and CRTC2/3-independent, it promotes VSMC mitosis [169]. PKC can directly act on CREB and phosphorylate its specific amino acid residues, leading to VSMC growth arrest [170, 171]. In addition, researchers have also discovered an interesting phenomenon that in VSMCs, activation of PPAR-γ induces the expression of LO enzymes (12-LO and 15-LO) and the secretion of LO products (12-HETE and 15-HETE), and these LO products can further upregulate the expression of PPAR-γ-2, forming a positive feedback amplification loop to enhance the activity and function of PPAR-γ [172].

Nitroolefin is a nitration product of unsaturated fatty acids. It activates the Keap1/Nrf2 signaling pathway in a dose-dependent manner, inducing Nrf2 nuclear translocation and increased ARE-driven transcriptional activity, leading to upregulation of VSMC cell cycle-dependent kinase inhibitor p27kip1 expression and proliferation inhibition [173].

Treatment with 200 μM palmitate promotes the contractile phenotype of VSMCs by up-regulating miR-22. This process is mediated by the transcription factor Ecotropic Virus Integration Site 1 (EVI1) [174]. miR-22 negatively regulates EVI1 by binding to the 3’-UTR of EVI1. Knockdown of EVI1 can mimic the inhibition of VSMC proliferation and migration by overexpression of miR-22 [175]. These findings highlight a complex relationship among miR-22, fatty acid metabolism, and VSMC phenotype. Future studies should aim to delineate the cellular and molecular contexts in which fatty acids exert contractile versus proliferative effects on VSMCs.

## VSMC amino acid metabolism

### Glutamate, glutamine and glutathione

Glutamine and glutamate are important free amino acids. The metabolism of glutamine starts with glutaminase deamination to produce glutamate and ammonia. Then glutamate is converted into α-ketoglutarate (α-KG), an intermediate of the TCA cycle, by glutamate dehydrogenase to compensate for the deficiency of glucose metabolism and OXPHOS [176]. The latest study found that glutamine and serine as anaplerosis of the TCA cycle increased in unstable plaques [5]. At the same time, single-cell RNA sequencing and microarray analysis found that glutamate metabolism-related enzymes Glutamate-Ammonia Ligase and Glutaminase (GLS) and glutamate receptor subunits GRIA1 and GRIA2 were highly expressed in VSMCs. The selective AMPA receptor NBQX inhibited the expression of VSMC contractile markers and inhibited proliferation [177].

Glutathione (GSH) is a major cellular antioxidant whose depletion disrupts redox homeostasis and promotes lipid peroxidation [178]. Glutathione peroxidase 4 (GPX4), a key inhibitor of ferroptosis, uses GSH as a cofactor to reduce lipid hydroperoxides to the alcohols and prevent lipid peroxides accumulation [179]. Bioinformatics studies have identified Yes-associated protein 1 (YAP1) as a potential key regulator of ferroptosis in VSMCs of unstable plaques. Further studies have found that YAP1 promotes the synthesis of Glu and GSH by regulating GLS, thereby increasing GPX4 expression and inhibiting ferroptosis in VSMCs [180]. GPX1 is also a member of the GPX family, in which the redox-sensitive selenocysteine amino acid participates in enzymatic reduction to maintain redox balance [181]. GPX1 deficiency leads to GSH accumulation and an increase in the GSH/GSSG ratio, inducing S-glutathionylation at Cys463 and Cys333 of SHP-2 and sustained activation of c-ros oncogene 1, stimulating VSMC proliferation and migration, and promoting vascular remodeling and endothelialization [182].

### Leucine metabolism

Leucine supplementation activates the SIRT1-Foxo1 pathway through the Slc3a2/Slc7a5 transporter, inhibiting the transformation of VSMC to a synthetic phenotype, vascular inflammatory response, and ROS generation during aging [183].

### Tryptophan metabolism

Tryptophan is an essential amino acid with two primary metabolic pathways: one involves sequential catalysis by tryptophan hydroxylase and arylalkylamine N-acetyltransferase to produce serotonin and melatonin; The second pathway involves conversion by tryptophan 2,3-dioxygenase (TDO) in the liver and IDO(indoleamine 2,3-dioxygenase 1) in other tissues (brain, gastrointestinal tract, liver) into the kynurenine pathway, where approximately 90% of tryptophan is degraded [184–186]. Multiple clinical studies indicate that decreased circulating tryptophan levels correlate with increased cardiovascular disease and coronary heart disease risk. Elevated metabolites such as kynurenine and 3-hydroxy-kynurenine predict adverse cardiovascular events in patients with end-stage renal disease and angina pectoris, and are closely associated with the progression of subclinical AS [187, 188]. IDO1 is the rate-limiting enzyme in tryptophan metabolism, and Kynurenine is an IDO1-mediated tryptophan metabolite [189]. The transformation of VSMC to an osteogenic phenotype was observed in IDO1^−/−^ApoE^−/−^ mice and Kynurenine supplementation promoted the assembly of the CUL4B-AhR E3 ubiquitin ligase complex by activating AhR, inducing RUNX2 ubiquitination and degradation, and inhibiting VSMC osteogenic reprogramming [190].

### Homocysteine (Hcy)

Hcy is an intermediate product of the methionine cycle and has become an independent risk factor for AS [191, 192]. Hcy can induce the VSMC phenotype to switch to anabolic or osteogenic types through multiple pathways such as autophagy, microRNA, and epigenetic regulation. Hcy treatment induces VSMC proliferation and migration in a dose-dependent manner, which is triggered by Hcy-mediated KLF4 inhibition and mTOR-mediated impairing autophagy. In KLF4-silenced VSMCs, autophagy was inhibited, while SMα-actin, SM-MHC, and mTOR expression levels were increased. Rapamycin (mTOR inhibitor) failed to reverse these changes, indicating a potential role between impaired autophagy mediated by the KLF4-dependent mTOR signaling pathway and the VSMC phenotypic remodeling [193].

S-adenosylhomocysteine (SAH) is also an intermediate metabolite of the methionine cycle and can be hydrolyzed into adenosine and Hcy by SAH hydrolase [194]. SAH accumulation and adenosine deficiency caused by SAH deficiency inhibits SIRT1-mediated H19 promoter hypoacetylation and DNMT3b-mediated hypermethylation through the AMPK pathway, inducing VSMC differentiation into H19-mediated Runx2-dependent osteoblasts to form atherosclerotic calcification [195]. In the ECs and VSMCs co-culture system, Hcy upregulated the levels of the PDGF through DNA demethylation in ECs, inducing VSMC proliferation and migration [196].

In addition, Hcy activated the PI3K/AKT/mTOR signaling pathway by inhibiting miR-145 expression, inducing VSMC proliferation, migration, and transformation to a synthetic phenotype. Overexpression of miR-145 reversed the above results [197]. S-propargyl cysteine, a Hcy derivative, can maintain the contractile phenotype of VSMC by upregulating miR-143-3p [198]. These findings suggest that targeting the miR-145/miR-143 axis may provide a potential strategy to prevent Hcy-induced VSMC phenotypic remodeling.

## Interactions among VSMC metabolic pathways and phasic dominance in phenotypic remodeling

During AS, VSMCs undergo dynamic transitions through multiple phenotypes. This phenotypic plasticity is not determined by a single signaling pathway but rather driven by the reprogramming and cross-regulation of multiple metabolic pathways. Different metabolic networks exhibit phasic dominance in VSMCs fate determination, forming crosstalk and compensatory mechanisms under specific pathological conditions. Dynamic crosstalk and compensatory mechanisms exist among the three metabolic pathways of VSMCs through shared metabolic nodes. Enhanced glucose metabolism promotes lipid synthesis and activates amino acid metabolism; disrupted lipid metabolism, in turn, negatively regulates glycolysis and amino acid utilization via ROS and ER stress, while amino acid metabolism provides compensatory substrates for impaired glucose and lipid metabolism during energy deficiency.

In early atherosclerotic plaques, inflammatory mediators and oxidative stress induce glucose metabolism reprogramming, shifting VSMCs from OXPHOS to aerobic glycolysis. Upregulation of key enzymes like GLUT1, HK2, and PFKFB3 provides abundant ATP and metabolic intermediates, supporting migration, proliferation, and ECM synthesis in synthetic VSMCs. Concurrently, glycolytic byproduct lactate promotes VSMC dedifferentiation by stabilizing HIF-1α, reinforcing the synthetic phenotype. During this phase, glucose metabolism dominates, providing the energy and carbon skeleton foundation for phenotypic shaping.

As atherosclerotic plaque formation and lipid deposition intensify, lipid abnormalities progressively emerge as key regulators of mid-to-late-stage phenotypic remodeling. Excessive ox-LDL and cholesterol loading induce VSMC expression of receptors like CD36 and LOX-1, leading to lipid phagocytosis and the formation of foam cells. At this stage, VSMCs not only lose contractile phenotype but also acquire macrophage-like and inflammatory characteristics, secreting factors such as IL-1β and MMPs that exacerbate plaque instability. Lipid accumulation induces mitochondrial dysfunction and excessive ROS production, further suppressing glycolysis and altering the NAD⁺/NADH ratio. This shifts metabolic balance toward lipid dependency, establishing a lipid-driven inflammatory phenotype.

In disease late stages or calcification phases, amino acid metabolism regulation progressively intensifies. Glutamine metabolism participates in the TCA cycle via α-KG and modulates histone demethylation, sustaining VSMC dedifferentiation while influencing osteogenic gene expression to drive osteoblastic-like transformation. Conversely, the tryptophan-kynurenine pathway regulates cellular inflammation and autophagy via AhR and mTOR signaling, exerting compensatory and homeostasis-maintaining effects that aid VSMCs survival under energy stress.

## Therapeutic strategies targeting metabolic reprogramming of VSMCs in AS

This section summarizes natural products, unlisted drugs, or marketed drugs that target glucose, lipid, and amino acid metabolism to inhibit VSMC phenotypic remodeling, stabilize plaques, and alleviate the progression of AS. (Table 1)

**Table 1:** Potential treatments target the metabolism of VSMC during the process of AS.

Drug or Intervention	Metabolism	Function	Model and Intervention	Mechanism
Dapagliflozin	glucose metabolism	Inhibition of osteogenic dedifferentiation of VSMCs	Dapagliflozin(5 mg/kg/d) 8w C57BL/6 mice underwent 5/6 nephrectomy followed by feeding with a high-phosphate diet Dapagliflozin (2.5 μM, 5 μM, 10 μM) 24 h HASMCs	Dapagliflozin → ↓ glucose → ↑ NAD⁺/NADH ratio → ↑ SIRT1 → deacetylation & degradation of HIF-1α → ↓ RUNX2, BMP2 ↑ α-SMA, SM22α → ↓ VSMC osteogenic dedifferentiation
linagliptin and empagliflozin	glucose metabolism	Inhibition of VSMC synthetic phenotype dedifferentiation	10 nM linagliptin or/and 100、500 或 1000 nM empagliflozin 4 d RASMCs	linagliptin+ empagliflozin→ ↓ intimal hyperplasia → ↓ VSMC proliferation
SRT1720	glucose metabolism	Inhibition of VSMCS senescence phenotype dedifferentiation	SRT1720 (10 μM) 1 h VSMCs from Sprague Dawley rats	SRT1720 (activates SIRT1) → ↑ PGC-1α deacetylation → improved mitochondrial function (↓ mtROS, ↑ mitochondrial DNA copy number, ↑ MMP, ↑ ATP synthesis, ↑ OCR) → ↓ VSMC senescence phenotype switching (↑ lamin B1, ↓ p53, ↓ 53BP1, ↑ BrdU-positive cells)
Metformin	glucose metabolism	Inhibition of osteogenic dedifferentiation of VSMCs	Metformin (500 μM) 1w VSMCs from Sprague Dawley rats	Metformin (activates AMPK) → ↓ PDK4 → ↓ oxidative stress (↓ ROS, ↑ SOD, ↓ MDA) → ↑mitochondrial function (↑ MMP, ↑ mitochondrial biogenesis genes, ↑ mitochondrial density) → ↓ apoptosis (↓ caspase-3/9, ↓ apoptotic cell ratio) →↓VSMC osteogenic dedifferentiation (↓ ALP, ↓ osteogenic genes, ↑ SM22α)
Intermedin	glucose metabolism	Inhibition of osteogenic dedifferentiation of VSMCs	Intermedin (100 ng/kg/h) 4w Sprague-Dawley rats	Intermedin→ ↑ cAMP/PKA signaling → ↓ GLUT1 → ↓ AGEs/RAGE signaling → ↓ VSMC calcification
Bempedoic acid	glucose metabolism	Inhibition of VSMC synthetic phenotype dedifferentiation	bempedoic acid (100 μM) HASMCs/MASMCs	Bempedoic acid (ACLY inhibitor) → ↓ ACLY expression → ↑ AMPKα/ACC signaling → inhibition of VSMC dedifferentiation and proliferation
Bisdemethoxycurcumin	glucose metabolism	Inhibition of VSMC-derived foam cells	·BDMC (10, 20, 40 mg/kg) 4w ApoE^−/−^mice ·BDMC(10、20、40 μM) 12 h VSMC	BDMC → ↓ PDK1/Akt/mTOR pathway → ↑ autophagy (↑ LC3B-II/LC3B-I ratio, ↑ Beclin-1, ↑ ABCA1, ↓ p62) → ↑ cholesterol efflux → ↓ lipid accumulation (↓ total cholesterol, ↓ triglycerides) → ↓ VSMC-to-foam cell transition
Dehydrodiisoeugenol	glucose metabolism	Inhibition of VSMC synthetic phenotype dedifferentiation	Dehydrodiisoeugenol (20 μM) PASMCs	Dehydrodiisoeugenol → ↓ mTOR activation → ↓ phosphorylation of p70S6K1 and 4EBP1 → ↓ HIF-1α and HK2 expression → ↓ glucose and lactate levels in VSMCs, inhibition of aerobic glycolysis → ↓ VSMC proliferation and migration
Sodium tanshinone IIA	glucose metabolism	Inhibition of VSMC synthetic phenotype dedifferentiation	Sodium tanshinone IIA (0, 1, 3, 10, 30 μM) 48 h VSMCs from Sprague Dawley rats	Sodium tanshinone IIA → ↑ AMPK activation (T172 phosphorylation) → ↑ p53 and p21 expression → ↓ Cyclin D1 expression → G0/G1 cell cycle arrest → ↓ VSMC proliferation STS → ↑ AMPK activation → ↓ NF-κB nuclear translocation → ↓ MMP-2 expression and activity → ↓ VSMC migration
Gualou-Xiebai herb pair and its active ingredients (albiflorin, apigenin, luteolin, kaempferol, 7,8-dihydroxyflavone, and hesperetin)	lipid metabolism	Inhibition of VSMC-derived foam cells	GLXB (3, 6, 12 mg/kg) 4w ApoE − /− mice GLXB medicated serum (5%, 10%, 15%) 48 h VSMCs from C57BL/6 mice	Gualou Xiebai Decoction and its active components→ ↓ P2RY12-PI3K pathway → ↑ lipophagy (↑ LC3II, ↓ p62, ↓ plin2) → ↓ VSMC-derived foam cell formation
10-Hydroxydec-2-enoic acid	lipid metabolism	Inhibition of VSMC inflammation	10-hydroxy-2-decenoic acid (1, 2, 4 mM) 1 h VSMCs from C57BL/6 mice	10-hydroxy-2-decenoic acid → ↓ TLR4 expression → ↓ SP1 and, expression → ↓ phosphorylation of ERK1/2, TAK1, and NF-κB p65 → ↓ TNF-α, IL-2, and IL-6 levels, ↑ IL-10 level → ↓ ROS generation, ↑ GSH and SOD levels → ↓ VSMC inflammatory response
Gallic acid	lipid metabolism	Inhibition of osteogenic dedifferentiation of VSMCs	gallic acid (20,30,40 μM) 48 h A7r5 cell	gallic acid → ↑ AMPK activation → ↑ eNOS phosphorylation → ↑ NO production → ↓ oleic acid-induced VSMC proliferation
Ganoderma lucidum spore powder and its derived triterpenes	lipid metabolism	Inhibition of osteogenic dedifferentiation of VSMCs	GLSP (1400 mg/kg/day) 12w LDLR^−/−^ mice ·GLSP-derived triterpenes (10 µM) 24 h HASMCs	GLSP → ↓ RUNX2 expression & nuclear translocation → ↓ VSMC osteogenic differentiation
ApoB ASO	lipid metabolism	Inhibition of VSMC-derived fibromyocytes and chondromyocytes	ApoB ASO (5 mg/kg per week) 4w Myh11-CreERT2 × tdTomato (tdT) reporter mice and Ldlr⁻/⁻ mice	ApoB ASO → ↑ ABCA1/ABCG1→ ↓ ApoB lipoproteins → ↓ NF-κB signaling → ↓ VSMC-derived fibromyocytes/chondromyocytes
Folic acid	amino acid metabolism	Inhibition of VSMC synthetic phenotype dedifferentiation	Folic acid (1μmol/L, 10μmol/L, 100μmol/L) 24 h VSMCs from Sprague Dawley rats	Folic acid → ↓ mTOR/P70S6K signaling → ↓ VSMC dedifferentiation Rapamycin (mTOR pathway inhibitor) → ↓ VSMC dedifferentiation MHY-1485 (mTOR pathway activator) → reverses the inhibitory effect of folic acid on VSMC dedifferentiation
Folic acid	amino acid metabolism	Inhibition of VSMC synthetic phenotype dedifferentiation	·Folic acid (75 μg/kg/d) 16w LDLR^−/−^ mice (C57BL/6 J background) ·Folic acid (20 μmol/L) 24 h MOVAS	Folic acid + Rapamycin →improved lipid metabolism (↓ TC, TG, LDL-C, VLDL-C; ↑ HDL-C) → ↓ mTOR/p70S6K signaling → ↓ oxidative stress (↑ SOD, ↑ GSH-Px, ↓ MDA) & ↓ inflammatory response (↓ IL-6, ↓ IL-1β, ↓ TNF-α) → ↓ VSMC dedifferentiation
β-aminoisobutyric acid	amino acid metabolism	Inhibition of VSMC synthetic phenotype dedifferentiation	BAIBA (3, 10, 30 μM) 48 h VSMCs	BAIBA → ↑ LKB1/AMPK/SIRT1 axis → ↓ VSMC inflammation (↓ phosphorylation and degradation of p65 NF-κB and IκBα; ↓ STAT3 phosphorylation) → ↓ VSMC oxidative stress (↓ NOX1, ↓ NOX4, ↓ phosphorylated p47^phox and membrane p47^phox; ↑ Nrf2 activation) → ↓ VSMC proliferation and migration

### Targeting glucose metabolism

Dapagliflozin is a member of the sodium-glucose co-transporter 2 inhibitor (SGLT2i) family, which can downregulate intracellular glucose levels and upregulate the NAD^+^/NADH ratio. The NAD^+^/NADH ratio can directly enhance the activity of the NAD^+^-dependent deacetylase SIRT1, thereby deacetylating and degrading HIF-1α, inhibiting RUNX2 and BMP2, and upregulating α-SMA and SM22α, inhibiting the VSMC osteogenesis and vascular calcification [199]. At the same time, SGLT2i combined with DPP-4 inhibitors can inhibit the VSMC proliferation in a dose-dependent manner and alleviate intimal formation after vascular injury [200]. SRT1720 is a synthetic compound with SIRT1 activation activity, which can improve oleic acid-induced VSMC mitochondrial respiratory and metabolic dysfunction by activating the SIRT1/PGC-1α pathway, promoting the cell senescence, and inhibiting the transformation of VSMC senescence phenotype [201]. Metformin promoted mitochondrial biogenesis by inhibiting PDK4, thereby suppressing VSMC osteogenesis [202].

Intermedin/Adrenomedullin-2 is a cardiovascular protective peptide derived from the ADM superfamily. It non-selectively binds to the calcitonin receptor-like receptor/receptor activity-modifying protein receptor complex and is involved in the arterial calcification [203]. The latest research found that Intermedin can activate the cAMP/PKA signaling pathway in a high-glucose environment to inhibit GLUT1 and inhibit VSMC osteogenesis [204].

Bempedoic acid is an ATP citrate lyase (ACLY) inhibitor that can activate the AMPKα/ACC signaling pathway by downregulating ACLY, inhibiting VSMC dedifferentiation and proliferation to alleviate intimal hyperplasia [205].

Bisdemethoxycurcumin (BDMC) from *Curcuma longa* inhibits PDK1/AKT/mTOR signaling in ox-LDL-stimulated VSMCs, promoting autophagy (increased LC3B-II/I ratio, Beclin-1, and ABCA1) and reducing dedifferentiation of VSMCs into foam cells [206]. Dehydrodiisoeugenol, a lignan derived from *Myristica fragrans Houtt* bark, inhibit aerobic glycolysis through the mTOR/HIF-1α/HK2 pathway to attenuate PDGF-BB-induced VSMC proliferation and migration [207]. In addition, dehydrodiisoeugenol can also activate SIRT1 to reduce Drp1 acetylation and increase Nrf2 expression to alleviate palmitate-induced VSMC mitochondrial damage and inhibit VSMC proliferation and migration by reducing oxidative stress and promoting autophagy [208, 209]. Sodium tanshinone IIA inhibits high glucose-induced VSMC proliferation and migration by activating the AMPK pathway and its downstream signaling pathways AMPK/p53/p21 and AMPK/NF-kB [210]. Collectively, these natural products regulate glucose metabolism to modulate VSMC phenotypic remodeling, providing new therapeutic opportunities for AS.

### Targeting lipid metabolism

Dysregulated lipid metabolism plays a central role in VSMC phenotypic remodeling and atherosclerotic plaque progression, making it an attractive target for therapeutic intervention. The herbal combination of Gualou and Xiebai can inhibit the P2RY12-PI3K signaling pathway, enhance LC3II expression in adipose tissue, reduce p62 and plin2 levels and reduce lipid droplets formation and foam cell derivation in VSMC by inhibiting lipophagy [211]. 10-Hydroxy-decan-2-enoic, a bioactive compound from royal jelly, dose-dependently inhibits the expression of TLR4 in AngII- or LPS-stimulated VSMCs and subsequently inhibits serine/threonine protein phosphatase 6 catalytic subunit (PPP6C), ERK1/2, and NF-κB p56. Molecular docking found that it competitively blocks TLR4–LPS binding through hydrogen bonds, unfavorable receptor-receptor interactions, or van der Waals forces, and subsequently reducing downstream inflammatory factors [212]. Gallic acid inhibits oleic acid-induced VSMC proliferation by regulating the AMPK-eNOS-FAS signaling [213]. Ganoderma lucidum spore powder and its derived triterpenes Ganoderic acid A, C6, G, and Ganodermanontriol enhance ABCA1/G1-mediated macrophage cholesterol efflux, suppress foam cells formation, inhibit RUNX2-mediated VSMC osteogenesis and stabilize plaques in LDLR^-/-^ mice by reducing necrotic core areas, increasing collagen content, and preventing calcification [214].

ApoB ASO is an antisense oligonucleotide drug targeting ApoB, which reduces the production of ApoB-lipoprotein in the liver by downregulating ApoB-100 gene expression and protein translation [215]. It also modulates VSMC abundance, phenotypes, and function by inhibiting NF-κB signaling, preserving the contractile phenotype, depleting VSMC-derived fibromyocytes and chondrocytes, and attenuating degenerative changes in atherosclerotic plaques [216]. Together, these natural products and targeted therapies highlight that modulating lipid metabolism and inflammatory signaling can effectively regulate VSMC phenotypic remodeling and improve plaque stability.

### Targeting amino acid metabolism

Folic acid is a key regulator of nucleotide synthesis and methylation reactions. It also cooperates with vitamin B12 to participate in the remethylation of Hcy and reduce the circulating Hcy levels [217]. Many experiments have shown that folic acid reduces Hcy and affects the magnesium ion homeostasis, oxidative stress, and inflammation of VSMCs, saving its phenotypic remodeling, with the mTOR/P70S6K pathway playing a central role in this process [218–220]. β-Aminoisobutyric acid, a natural non-protein β-amino acid, activates the LKB1/AMPK/SIRT1 axis, promotes NF-κB p65 phosphorylation and nuclear translocation, delays IκBα phosphorylation and degradation, alleviates VSMC inflammation, and activates Nrf2 to relieve oxidative stress, collectively inhibiting VSMC proliferation and migration [221]. These findings highlight the potential of folic acid and β‑aminoisobutyric acid to regulate VSMC phenotype by balancing amino acid metabolism and inflammation.

## Emerging techniques identify VSMC dedifferentiation trajectories

The phenotypic plasticity of VSMCs is central to vascular remodeling and atherosclerotic progression. In recent years, the rapid advancement of single-cell and spatial transcriptomics technologies has provided unprecedented spatiotemporal resolution for deciphering the dedifferentiation trajectories of VSMCs. (Table 2)

**Table 2:** Application of emerging techniques in studying VSMC phenotypic remodeling.

Technology	Application	Related research	Advantages/Limitations
Single-cell transcriptomics	**Live-seq**: Maintains cell viability during RNA extraction using FluidFM, enabling coupling of basal transcriptome with downstream phenotypes.	Transcriptome of cells before and after differentiation to directly map the trajectory of cells [222]	Transforms conventional scRNA-seq from an endpoint approach into a temporal analytical framework
	**CITE-seq**: Profiles both transcriptome and surface epitopes by sequencing at single-cell resolution.	Integrated multi-sample single-cell datasets to identify fibromyocytes as a vascular-specific cell population [223]	Integrates all available datasets to achieve coherent automated cell-type annotation, optimal experimental design, and bulk deconvolution.
	**FindMarkers**: Identifies marker genes for each cluster to define major cell types.	VSMC were distinguished according to surface markers [224]	Determines the temporal trajectory of VSMC dedifferentiation during vascular remodeling and atherosclerosis.
	**AddModuleScore**: Scores and evaluates senescence-associated gene modules.	Evaluated senescence-associated gene signatures within VSMC clusters [37]	Determines the temporal trajectory of VSMC dedifferentiation during vascular remodeling and atherosclerosis.
	**Reactome/Enrichr**: Conducts pathway enrichment analysis.	Identified key transcriptional factors driving the transition from contractile SMCs to stem-like SEMs [37]	Determines the temporal trajectory of VSMC dedifferentiation during vascular remodeling and atherosclerosis.
Spatial transcriptomics	**Slingshot**: Performs pseudotime trajectory analysis to infer cell differentiation pathways.	Determined spatial migration trajectory of VSMCs from the media to the lumen, transitioning through a myofibroblast-like intermediate state and differentiating into fibromyocytes within plaques [225]	Determines the spatial trajectory of VSMC dedifferentiation during vascular remodeling and atherosclerosis.
Lineage tracing	**Myh11**	**Myh11-DreERT2×Lgals3-Cre**: Dual recombinase (Dre-rox and Cre-loxP) lineage tracing system used to track VSMC transition toward Lgals3⁺ phenotypes [231]	·High precision due to independent Dre-rox and Cre-loxP systems, minimizing crosstalk. ·Enables tracking of SMC-to-Lgals3⁺ transition. Distinguishes untransformed (tdTomato⁺) from transformed (eGFP⁺) SMCs.
		**Myh11-CreER**^**T2**^: Myh11-driven labeling of VSMCs for tracking dedifferentiation, migration, inflammation, and interactions with macrophages and fibroblasts [226–228]	·CreERT2 insertion on Y chromosome limits studies to male mice. ·Labeling efficiency low, with possible off targets (ECs, macrophages, or stem cells)
		**Myh11-CreERT2 RAD**: Myh11-based VSMC labeling model for studying sex effects on dedifferentiation trajectories [229]	·Applicable to both sexes. ·Improved labeling efficiency (>95% in aorta and carotid artery).
		**Myh11-CreNLS**: Constitutive system for embryonic or early postnatal SMC lineage tracing [230]	·Applicable to both sexes. ·NLS enhances nuclear localization and recombination efficiency.
	**Tagln/SM22α**	**Tagln-Cre**: SM22α-driven labeling used to study VSMC senescence, proliferation, and calcification; also labels myeloid cells [232–235]. **Tagln-CreKI**: Knock-in line for adult SMC localization in arteries, veins, and visceral organs [236]	·Low labeling efficiency, co-labels bone marrow–derived cells (neutrophils, monocytes, macrophages). ·Expressed appropriately in adult SMCs but silent in embryonic SMCs.
	**Acta2**	**Acta2-Cre/Acta2-Cre-ER**^**T2**^: Used to trace VSMC dedifferentiation during vascular remodeling [237]	·Low labeling efficiency, with possible off targets (myofibroblasts and myoepithelial cells)
	**Itga8**	**Itga8-Cre/Itga8-CreER**^**T2**^: Applied to vascular reconstruction studies [238, 239]	·Prevents myeloid cell leakage and avoids lethal visceral myopathy seen in Myh11-CreERT2 lines.

### Single-Cell and Spatial Transcriptomics

The application of single-cell sequencing technologies enables researchers to reveal the molecular dynamics of VSMC transformation at the single-cell level. Live-seq utilizes FluidFM to extract RNA while maintaining cell viability, allowing the analysis of transcriptional differences before and after differentiation without cell disruption. This transforms traditional endpoint scRNA-seq into a temporal dynamic analysis framework [222].

CITE-seq further simultaneously measures transcriptomes and cell surface epitopes at single-cell resolution. After integrating multi-cohort data, researchers identified fibromyocytes as a vascular-specific population and established reproducible surface markers [223]. Combining FindMarkers and AddModuleScore analyses enabled the identification of marker genes for each cluster and the evaluation of aging-related gene modules. Results revealed fibromyocytes enriched with aging-related gene, suggesting synchronized cell cycle arrest and phenotypic conversion [37, 224]. Enrichment analysis using Reactome and Enrichr further uncovered key transcription factor networks driving the conversion of contractile VSMC into stem-like smooth muscle-like cells [37]. Spatial transcriptomics combined with Slingshot pseudo-time analysis revealed the migration trajectory of VSMC within the vascular wall: VSMC radially migrate from the media to the intima, differentiate into plaque-associated fibrocytes after passing through a myofibrillar intermediate state, and exhibit a spatial trajectory highly correlated with the gene expression gradient of ECM remodeling. This reflects the spatial continuity and functional heterogeneity of VSMC phenotypic remodeling [225].

### Lineage tracing technology

Lineage tracing provides direct biological evidence to validate single-cell results. The traditional Myh11-CreER^T2^ model is commonly used to track VSMC migration, inflammation, and interactions with immune cells. However, its CreER^T2^ insertion into the Y chromosome limits its application to male individuals, exhibits low labeling efficiency, and results in non-specific labeling of cells such as endothelial cells or macrophages [226–228]. The improved Myh11-CreER^T2^ RAD model is applicable in both male and female individuals, achieving >95% labeling efficiency [229]. Concurrently, the NLS-enhanced Myh11-CreNLS system improves nuclear localization and recombination efficiency, making it suitable for lineage tracing during embryonic and early developmental stages [230]. The Myh11-DreERT2×Lgals3-Cre dual recombination system achieves highly specific tracing via independent Dre-loxP and Cre-loxP pathways, confirming that Lgals3⁺ cells originate from in situ conversion of VSMCs rather than myeloid infiltration [231].

Tagln-Cre and its knock-in variant Tagln-CreKI have been used to study VSMC aging, proliferation, and calcification. However, their labeling of both myeloid and bone marrow-derived cells limits their specificity [232–236].

Acta2-Cre and Acta2-CreERT2 models can track VSMC dedifferentiation during vascular remodeling, but exhibit low labeling efficiency and prone to mislabeling myofibroblasts [237].

The Itga8-Cre/Itga8-CreERT2 model avoids myeloid cell leakage and does not induce the lethal visceral myopathy commonly seen in Myh11-driven models, making it an ideal tool for studying VSMC sex differences and remodeling processes [238, 239].

## Conclusions and Prospects

In this review, we highlight the important role of VSMCs in the development of AS, with a particular focus on how their metabolic reprogramming and phenotypic changes contribute to plaque formation and stability. The connection between cellular metabolism and VSMC phenotype presents potential opportunities for therapeutic intervention. Moving forward, research should aim to clarify the molecular mechanisms underlying VSMC remodeling and explore ways to target these processes. Modulating metabolic pathways may offer new strategies for stabilizing plaques and slowing disease progression. A better understanding of VSMC metabolism could ultimately lead to more effective approaches for the prevention and treatment of cardiovascular disease.
